# Disease burden and unmet need for acute allergic reactions – A patient perspective^☆^

**DOI:** 10.1016/j.waojou.2024.100896

**Published:** 2024-03-30

**Authors:** Emelie Andersson, Sofia Löfvendahl, Sara Olofsson, Karin Wahlberg, Leif Bjermer, Göran Tornling, Christer Janson, Jonas Hjelmgren

**Affiliations:** a The Swedish Institute for Health Economics (IHE), Lund, Sweden; b Department of Respiratory Medicine and Allergology, Lund University, Lund, Sweden; c Respiratory Medicine Division, Department of Medicine Solna, Karolinska Institutet, Stockholm, Sweden; d Department of Medical Sciences: Respiratory, Allergy and Sleep Research, Uppsala University, Uppsala, Sweden

**Keywords:** Allergy, Acute allergic reactions, Disease burden, Corticosteroids, Anaphylaxis

## Abstract

**Background:**

Acute allergic reactions (AARs) occur shortly after exposure to an allergen, and the severity is on a continuum. Systemic corticosteroids (CS) are mainstay treatment of moderate to severe AARs, whereas those at risk of the most severe AARs (ie, anaphylaxis) are also recommended prescription of epinephrine autoinjectors. There is limited research on the impact of AARs not fulfilling the criteria for anaphylaxis. We have characterized a sample with a history of moderate to severe AARs and evaluated their self-reported disease burden (ie, daily life impact, anxiety, and treatment impediments).

**Methods:**

Survey study of adults with experience of AARs treated with CS. Participants recruited from a web-based panel and using social media were asked to complete a questionnaire related to their allergy and experience of AARs. The results were summarized for the whole sample and across subgroups with and without prescription of epinephrine.

**Results:**

The final study sample included 387 participants (80% women, mean age 41), of which 129 (33%) had at some point been prescribed epinephrine. The most common symptoms were respiratory (80%) and skin (78%) manifestations, and the mean (standard deviation, SD) self-rated severity score (scale from 0 [very mild] to 10 [very severe]) of the most recent AAR was 6.1 (2.0). More than 80% had experience of AARs interrupting daily activities and 50% of AARs that had limited work/studies or participation in leisure activities. Most of the respondents reported some degree of anxiety related to AARs and 43% had feared for their lives. Moreover, difficulties swallowing allergy medicine at an AAR was experienced by 26% and not having the medicine available when needed by 66%. Participants with prescription of epinephrine experienced more severe AARs than those without such prescription (mean [SD] severity 6.8 [2.1] vs 5.8 [1.8], p < 0.0001); however, also those without epinephrine prescription reported considerable anxiety and impact on daily life and to a similar degree as those with prescription.

**Conclusions:**

In this sample, subjects with experience of AARs treated with CS showed a considerable disease burden with anxiety and interruption on daily life, as well as problems related to access to, and swallowing of, medication. Although respondents with epinephrine prescription had more severe disease, a high disease burden was also evident among those without epinephrine. The study increases the knowledge of people with moderate to severe AARs, a patient population that has previously been underrepresented in the research literature.

## Introduction

Allergic disease is on the rise globally with the highest prevalence reported for high-income countries.^1^^,^^2^ In Sweden, it has been estimated that more than 30% of the population suffer from some type of allergy.^3^

An allergic reaction can affect different organ systems (eg, circulatory, gastrointestinal, and respiratory systems) and manifests with a range of symptoms. In acute allergic reactions (AARs), symptoms arise shortly after the exposure but may be more delayed in food-allergies.^4^ The severity of AARs is a continuum from mild itching of the eyes, nose, mouth, and throat to the potentially life-threatening highest grade of anaphylaxis (ie, anaphylactic shock) with symptoms on airways and the cardiovascular system.^5^ Due to complex clinical manifestations, unclear definitions, and overlapping symptoms, the distinction between anaphylaxis and less severe AARs is not always clear.^5^

Although there is a lack of consensus treatment guidelines for AARs in general, systemic corticosteroids (betamethasone, dexamethasone, or prednisolone) in combination with antihistamines appear to be the mainstay treatment for moderate to severe AARs not fulfilling the criteria for anaphylaxis (eg, symptoms such as urticaria, angioedema, and nausea),^6^^,^^7^ whereas only antihistamines are generally recommended for mild AARs. In contrast, the first-line treatment for anaphylaxis is intramuscular epinephrine; antihistamines and systemic (oral) corticosteroids may be given as second line. Patients at risk of anaphylaxis are recommended prescriptions for epinephrine autoinjectors for self-treatment.^8^^,^^9^

Most research on disease burden and patient perspective of AARs have focused on anaphylaxis, and there is an apparent lack of studies evaluating the wider population suffering from AARs. Subjects with food allergy and those who have experienced an anaphylactic reaction have reported reduced quality of life and a high degree of anxiety related to fear of potentially fatal allergic reactions, concerns regarding disease management, and lack of control.^10^^,^^11^ However, it is unclear whether such concerns are limited to anaphylaxis, or if they also apply for patients with moderate to severe non-anaphylactic allergic reactions.

The aim of this study was to characterize and evaluate disease burden among patients with prescription of corticosteroids for treatment of moderate to severe AARs. The population included subjects both without and with prescription for epinephrine. The latter group is likely to have experienced more severe AARs including anaphylaxis. Treatment with epinephrine also involve a different treatment regimen. Therefore, we hypothesized that these groups could show differences in how they experienced impact on daily life, anxiety related to AARs and treatment impediments.

## Materials and methods

### Study design and recruitment

The study was cross-sectional and used a web-based survey. Study questions were developed in cooperation with allergologists and aimed to capture a broad picture of the disease burden. A visual analogue scale (VAS) was used as numeric rating scale, which is commonly applied within different diseases areas including allergy.^12^ The questionnaire consisted of 2 parts. The first part included questions about patients' characteristics, treatment, disease burden (eg, symptom severity, disruption of daily life, and anxiety), and unmet treatment need. In the second part, the value of attributes related to the administration mode of corticosteroids (mouth dissolving film versus corticosteroid tablets), was investigated using a willingness-to-pay method by applying a Discrete Choice Experiment approach. This article reports results from the first part of the questionnaire only.

The respondents were identified from a database (web-based panel) mainly used for recruitment to clinical trials, where approximately 3000 persons (February 1, 2022) had stated that they have some form of allergy and therefore received information about this study. In addition, a national, study-specific advertising campaign was published on social media platforms. The recruitment and data collection started July 1 and ended August 15, 2022. All data were anonymized, and the study was approved by the Swedish Ethical Review Authority (Dnr 2022-02147-01). Informed consent was collected from all participants and only complete (finalized and submitted) responses were used in the analysis.

### Study population

The study population included people with self-reported allergy and ever corticosteroid treatment for AARs. The inclusion criteria for participating in the study were:

• Eighteen years or older
• Self-reported allergic problems
• Experience of an AAR that required the use or prescription of oral corticosteroids

AARs can cover a broad range of symptoms and severity grades and there is no clear definition. In this study, having used or being prescribed corticosteroids for acute allergic reactions (as opposed to preventive treatment of pollen allergy) were the criteria for having experienced an AAR of moderate to high severity. The population was divided into 2 subgroups based on whether they had received a prescription for epinephrine (Question: Have you been prescribed an epinephrine autoinjector? Alternatives for answers: Yes/No/Do not know). The subgroup without prescription for epinephrine is hereafter referred to as “corticosteroid (CS)-only” and the subgroup with prescription for epinephrine as “CS + epinephrine autoinjector (EAI)”. However, it should be noted that respondents in both groups may have had other medical treatments for their AARs such as antihistamines or bronchodilators. Respondents providing the answer “do not know” were included in the group “CS-only”.

### Study outcomes and variables

The participants were provided information about the study via e-mail after which they could provide consent electronically and access the questionnaire. The participants were first asked to answer the screening questions for validation of the inclusion criteria. Those who passed the screening were provided the full questionnaire which covered general background questions (sex, age, income) and questions related to allergy. These included questions on allergic problems in general and attributes of AARs (types, symptoms, treatment, and severity) as well as questions related to disease burden of AARs including impact on daily life, anxiety, and treatment impediments. The questions represented in the results of this study, and the corresponding answer alternatives, are provided in Supplemental Table S1. To simplify interpretation and group comparisons of responses on numeric rating scales (NRS; ie, questions concerning reaction severity and security/anxiety with rating scores of 0–10), scores were combined into 3 levels: 0–3, 4–7, and 8–10. The uncombined NRS scores are presented in Supplemental Material.

### Statistical analysis

Results from the questionnaire were summarized using standard descriptive measures including number and proportion for categorical variables, and mean, standard deviation, or median and interquartile range (IQR) for continuous variables and for questions with answers referring to intervals.

All analyses were performed for the whole study population, and separately for respondents with and without prescription of epinephrine. Comparisons between these subgroups for continuous variables were performed by Student's t-test on the equality of means for normally distributed variables and Wilcoxon rank-sum (Mann-Whitney) test on the equality of medians for non-normally distributed variables. The distribution of categorical data across subgroups was explored by the Pearson's chi square test of independence. Detailed statistical output data is presented in Supplemental Material, Supplemental Table S2. Data were analyzed using STATA MC version 14.2.

## Results

### Demographic and socioeconomic characteristics of final study sample

The survey was sent to 830 people and was completed by 426 eligible respondents. Of these, 39 who stated that they had allergy only to pollen were excluded from analyses (See Supplemental Figure S1 for flowchart of inclusion). This exclusion was based on the assumption that people with allergy to pollen mainly use corticosteroids for preventive treatment of allergic symptoms (as opposed to acute treatment in moderate to severe AARs). The exclusion of respondents with allergy to pollen only was supported by a sub-analysis showing that this group differed significantly from the rest of the study sample in several areas including symptom manifestation (smaller proportion with specific symptoms), treatment (smaller proportion treated with epinephrine and fewer corticosteroid tablets taken at the most recent reaction), and daily life disruption (smaller proportion having experienced interruption or inability to carry out daily activities) (Supplemental Table S3).

The final study sample of eligible respondents was 387, of which 129 (33%) had at some point been prescribed epinephrine (Supplemental Figure S1). Mean (min, max) age of the study population was 41 (18, 74) years and 80% were women (Table 1). The majority (65%) had university education and 70% were working full or part time. The subgroups CS-only and CS + EAI were similar in terms of demographics and socioeconomic characteristics, with the exception that CS + EAI had a larger proportion of respondents with a high level of education (p = 0.001).

**Table 1:** Demographic and socioeconomic characteristics of the whole study sample and for subgroups separately.

	ALL N = 387	SUBGROUPS	
		CS-only n = 258	CS + EAI n = 129
**Sex**			
Females, n (%)	308 (80)	204 (79)	104 (81)
**Age**			
Years, mean (SD)	40.9 (13.7)	40.7 (13.7)	41.3 (13.8)
**Level of education, n (%)**			
Compulsory school	13 (3)	11 (4)	2 (2)
Upper secondary school	105 (27)	79 (31)	26 (20)
University <3 year	59 (15)	41 (16)	18 (14)
University ≥3 years	193 (50)	123 (48)	70 (54)
Other	17 (4)	4 (2)	13 (10)
**Work force participation, n (%)**			
Working (full time or part time)	272 (70)	181 (70)	91 (71)
Other (retired, student, on sick leave)	113 (29)	75 (29)	38 (29)
No answer	2 (<1)	2 (<1)	0 (0)

CS: corticosteroids; EAI: epinephrine auto injector; SD: Standard deviation.

### Allergy attributes and treatment

#### Types of allergy and symptoms of AARs

**Fig. 1:**
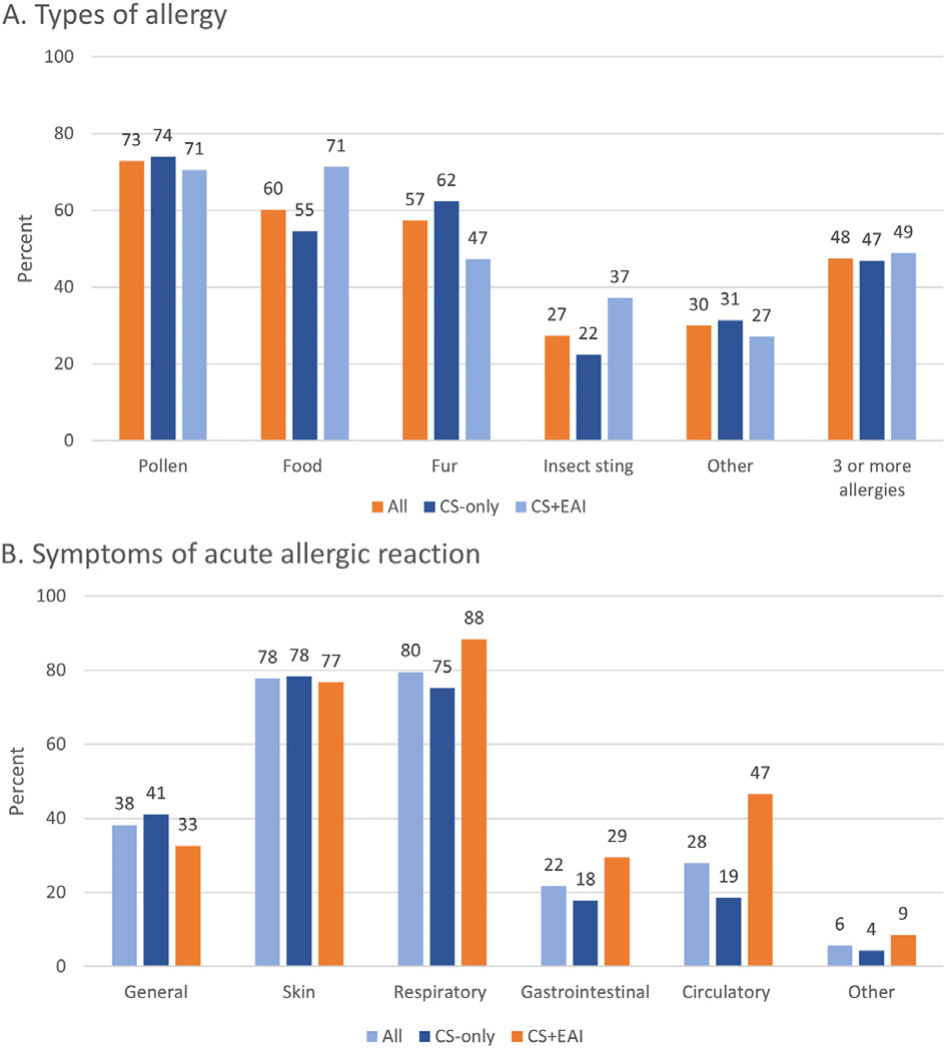
Types of allergy (Panel A) and affected organ systems (Panel B) of AARs in the whole study sample and in subgroups representing respondents with and without prescription for epinephrine. The figure shows the proportion of patients within each group with the specific symptoms/types of allergies. Note that 1 patient could have several types of allergies and symptoms. AAR: acute allergic reaction; CS: corticosteroids; EAI: epinephrine autoinjector.

Pollen allergy was the most common type of allergy in the whole sample and approximately half of the respondents had 3 or more types of allergies. The most common symptoms were respiratory symptoms (experienced by 80%), followed by skin (78%) and circulatory (28%) symptoms (Fig. 1).

Between subgroups, the CS + EAI subgroup had a higher proportion with allergy to food (71% vs 55%, p = 0.002) and insects stings (37% vs 22%, p = 0.003), whereas fur allergy was more common in CS-only (62% vs 47%, p = 0.006) (Fig. 1). In terms of symptoms, the subgroup CS + EAI had a higher proportion of respondents with respiratory (88% vs 75%, p = 0.002), gastrointestinal (30% vs 18%, p = 0.013), and circulatory symptoms (47% vs 19%, p < 0.0001). General symptoms (eg fatigue and anxiety) were somewhat more common in the CS-only group (41% vs 33%), but the difference was not significant (p = 0.12). Moreover, respondents in the CS + EAI subgroup had experienced reactions involving symptoms from more organ systems than those in CS-only (median number of organ systems affected 3 vs 2, p < 0.001).

#### Treatment of AARs

In the whole sample, the median of number of AARs treated with corticosteroids over the preceding year was 1 (Table 2), and 8 respondents (2%) reported more than 10 reactions. In their most recent AAR, the majority (67%) had taken between 11 and 15 corticosteroid tablets and nearly 40% had also at some point been treated with epinephrine for an AAR.

**Table 2:** Treatment experiences and severity of AARs.

	All n = 387	Subgroups	
		CS-only n = 258	CS + EAI n = 129
**Number of AARs treated with corticosteroid during the last year**			
Median (IQR)	1 (0, 2)	1 (0, 2)	1 (0, 3)
**Number of corticosteroid tablets taken at the last AAR? n (%)**			
Less than 5	7 (2)	6 (2)	1 (1)
5–10	130 (34)	112 (43)	18 (14)
11–15	131 (67)	81 (31)	50 (39)
16–20	94 (24)	47 (18)	47 (36)
More than 20	25 (6)	12 (5)	13 (10)
**At any time treated with epinephrine injection for an AAR**^a^**, n (%)**			
Yes	152 (39)	57 (22)	95 (74)
No	196 (51)	166 (64)	30 (23)
Do not know	39 (10)	35 (14)	4 (3)
**Severity of the most recent AAR**^b^**, n (%)**			
NRS 0–3 (“mild”)	31 (10)	27 (10)	10 (8)
NRS 4–7 (“moderate”)	270 (70)	195 (76)	75 (58)
NRS 8–10 (“severe”)	80 (21)	36 (14)	44 (34)

AAR: acute allergic reaction; CS: corticosteroids; EAI: epinephrine autoinjector; SD: Standard deviation; IQR: interquartile range; NRS: numeric rating scale.

a The question was “Have you been treated with epinephrine for an AAR” which gives different information than the question used for defining subgroups by covering treatment with epinephrine also for those without a prescription such as in emergency care.

b Levels based on an NRS from 0 (very mild) to 10 (very severe) were combined as indicated. The uncombined scores are presented in Supplemental Figure S2.

There was no significant difference between subgroups in the number of AARs treated with corticosteroids (Table 2); however, respondents in the CS + EAI group had taken more corticosteroid tablets than those without epinephrine at their most recent reaction (Table 2). The proportion of respondents who had been treated with epinephrine for an allergic reaction was considerably higher in the CS + EAI subgroup (74%) compared to CS-only (22%) (p < 0.0001).

#### Severity of AARs

On the NRS scale from 0 (very mild) to 10 (very severe), the mean (SD) perceived degree of severity of the most recent AAR was 6.1 (2.0) for the whole study sample and most respondents (70%) rated the severity with scores in the range of 4–7 (ie, “moderate” severity) (Table 2).

Respondents in the CS + EAI subgroup reported higher mean severity compared to respondents without epinephrine (6.8 [2.1] vs 5.8 [1.8], p < 0.0001). In CS + EAI, approximately one third graded the severity of their last acute reaction as 8 to 10 (ie “severe”) compared with 14% in CS-only (Table 2). In the CS-only subgroup, 81% reported a severity grade of 5 or more. Moreover, a higher proportion (85 vs 62%, p < 0.0001) of respondents in the CS + EAI subgroup reported that they had used emergency care due to an AAR compared with CS-only. Among those reporting emergency care, the average number (SD) of emergency care visits were 2.0 (2.5) for CS + EAI and 1.4 (1.1) for CS-only.

### Disease burden

#### Impact on daily life

**Fig. 2:**
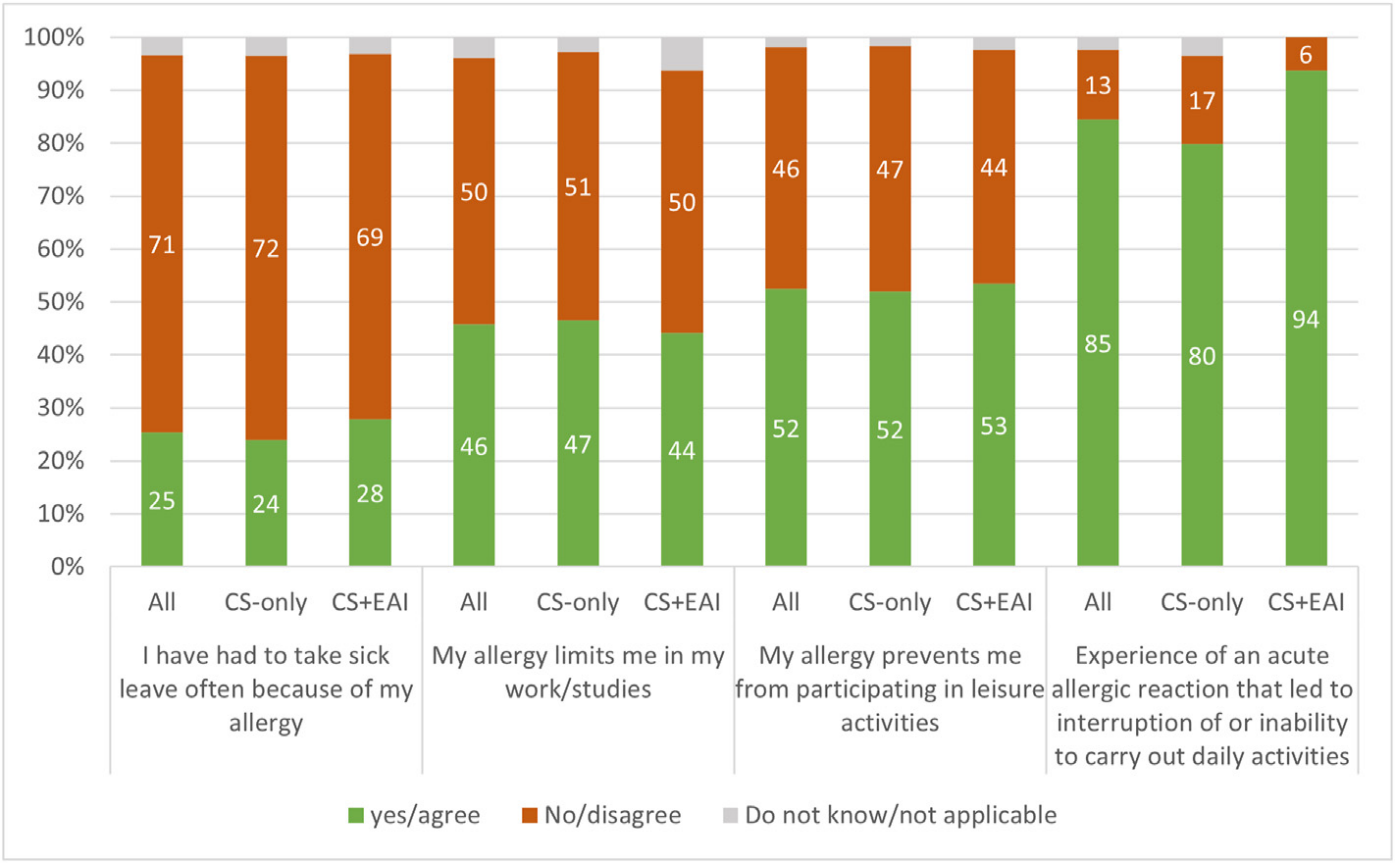
Results from questions concerning impact on daily life for the whole study sample and for subgroups (ie, respondents with and without prescription of epinephrine) separately. Agree covers the two answers “agree completely” and “agree to some extent”. Disagree covers the two answers “disagree completely” and “disagree to some extent”. For question with more than 3 alternative answers, results for all alternatives are presented in Supplemental Figure S3. CS: corticosteroids; EAI: epinephrine autoinjector.

Questions regarding impact on daily life revealed that a large proportion (87%) of respondents had experienced an AAR that had interrupted daily activities (Fig. 2). Limitations in work/studies or leisure activities due to allergy was reported by approximately half of the respondents and one quarter had been taking sick leave due to their allergy.

The experience of limitations related to daily activities was most common in the subgroup CS + EAI (94 vs 83%, p = 0.002). For questions concerning sick leave, work/studies and leisure activities, results were similar between subgroups (Fig. 2).

#### Anxiety

**Fig. 3:**
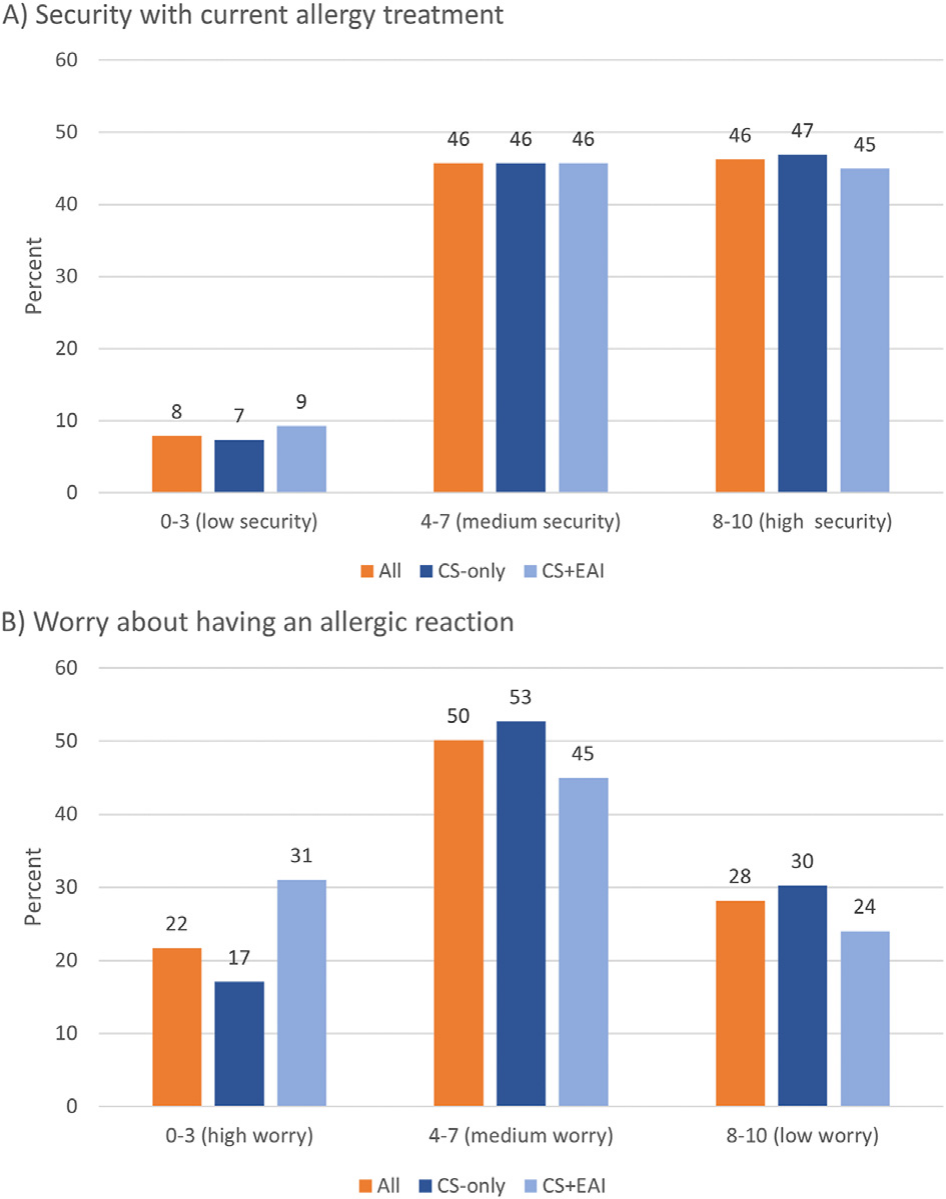
Results from questions concerning perceived security with medication (Panel A) and worry of having an AAR (Panel B). The degree of security/anxiety was graded on a numeric rating scale from 0 to 10 where 0 represents very worried/very insecure and 10 not worried at all/very secure. The figure shows the proportion of respondents within each group that have reported a certain grade. Results are presented for the whole study sample and for subgroups (ie, respondents with and without prescription of epinephrine) separately. To simplify interpretation and between group comparison, the scores 0–3, 4–7 and 8–10 were combined. Uncombined scores are presented in Supplemental Figure S4.

Respondents’ grading of their perceived degree of security with current treatment and worry about having an allergic reaction on an NRS scale from 0 to 10 (10 representing highest degree of security/lowest degree of worry) is presented in Fig. 3. In the whole study sample, the mean (SD) score was 7.1 (2.3) for sense of security with current allergy treatment and 5.7 (2.6) for worry about having an allergic reaction. More than 70% scored their worry in the range of 0–7. Moreover, 43% of the respondents reported that they had experienced an AAR that made them fear for their lives.

There was no difference in perceived security with allergy treatment between subgroups (p = 0.827). In terms of worry related to having an allergic reaction, respondents in the CS + EAI subgroup reported a lower mean (SD) score (5.1 [2.7] vs 6.01 [2.44], p = 0.002) than those in CS-only, indicating higher degree of worry. Moreover, in CS + EAI, nearly one-third reported their worry as 3 or less (ie, high degree of worry), compared with 17% for respondents in CS-only (Fig. 3). The proportions that had experienced AARs making them fear for their lives were 70% in CS + EAI and 29% in CS-only (p < 0.001).

#### Treatment impediments

One-quarter of all respondents had experienced difficulty in swallowing their allergy medicine when having an AAR, and two-thirds of the respondents had experienced a situation of not having their allergy medicine available when needed for an AAR (Table 3). These results were similar between subgroups. Among those with experience of not having their medicine available, the main reason, stated by 75% of the whole study sample, was being away from home without the allergy medicine.

**Table 3:** Results from questions concerning treatment impediments presented for the whole study sample and for subgroups separately.

		Subgroups	
	All n = 387	CS-only n = 258	CS + EAI n = 129
**Experience of difficulty swallowing allergy medicine when having an AAR, n (%)**			
Yes	100 (26)	57 (22)	43 (33)
No	271 (70)	190 (74)	81 (63)
Do not know	16 (4)	11 (4)	5 (4)
**Experience of not having the allergy medicine immediately available when needed for an AAR, n (%)**			
Yes	255 (66)	173 (67)	82 (64)
No	119 (31)	76 (29)	43 (33)
Do not know	13 (3)	9 (3)	4 (3)
**Reason(s) for not having allergy medication available when needed, n (%)**	**N=255**	**N=173**	**N=82**
Not bought/collected the allergy medicine	55 (22)	35 (20)	20 (24)
Away from home without the allergy medicine	191 (75)	137 (79)	54 (66)
Other	50 (20)	26 (15)	24 (29)

AAR: acute allergic reaction; CS: corticosteroids; EAI: epinephrine autoinjector.

## Discussion

In this study, we have characterized a sample with corticosteroids prescribed for treatment of AARs. The study sample represented a range of allergy types and symptoms. The results increase the understanding of moderate to severe AARs and how they are experienced by affected individuals, a topic that is scarcely represented in the published literature. They also provide important information on how such reactions are treated and whether treatment is associated with impediments.

A large proportion of the respondents reported that they had experienced severe AARs which had an impact on daily life in the form of interrupted daily life activities (85%), contributed to anxiety, and made them fear for their lives (43%). Moreover, only one-third of the respondents had a prescription for epinephrine, indicating that corticosteroids, with or without antihistamines, is the predominantly prescribed treatment for people with moderate to severe AARs. We also identified that many respondents had experienced impediments related to treatments for their AARs including difficulty in swallowing their allergy medicine and not having their allergy medicine available when needed. These impediments, which may have contributed to more severe reactions and anxiety among study participants, highlights an unmet treatment need for more accessible and easily administered treatment options among people with AARs.

In addition to analyzing the sample as a whole, we also compared 2 subgroups based on previous prescription for epinephrine, assuming that those with such prescription represents respondents with a more severe allergy and/or history of a very severe AAR. A prescription for epinephrine may also indicate that the person has experienced an anaphylactic reaction or been considered at risk of anaphylaxis. Most literature describing AARs is restricted to anaphylactic reactions, where epinephrine is recommended in treatment guidelines.^8^^,^^9^ The separate analyses on participants without EAI therefore give an opportunity to describe allergy and AARs in a less severely affected population and fill in some of the knowledge gaps in terms of disease burden for this patient category. We also hypothesized that the prescription for epinephrine could influence how the participants experienced their AARs, for instance in terms of anxiety and interruption of daily life. In addition, it could influence the treatment experience.

As expected, the subgroup analyses showed that the 30% of respondents with a prescription for epinephrine experienced more severe AARs than those without epinephrine. The respondents with epinephrine had more types of symptoms, higher perceived severity grade, more emergency care, and more medical treatment (ie, more corticosteroid tablets taken at the most recent reaction and higher proportion treated with epinephrine). In particular, the severity of allergic reactions among respondents with epinephrine was evident from the high proportion (70%) reporting that they had experienced reactions making them fear for their lives. However, the results also indicated a considerable disease severity among the respondents without prescribed epinephrine; in this subgroup >80% reported a severity grade of 5 or more (on a severity scale from 0 to 10), 62% had required emergency care, 22% had been treated with epinephrine, and 29% had feared for their lives. Moreover, in terms of disease burden, the vast majority reported anxiety related to AAR and for most aspects concerning impact on daily life activities, the results were similar to those with epinephrine prescription.

The size of the subgroup without epinephrine, and the relatively high degree of symptom severity and disease burden experienced by these respondents, is particularly interesting considering the apparent underrepresentation of patients with non-anaphylactic allergic reactions in the literature and the lack of established consensus treatment guidelines for this type of reactions. The findings are in line with data from the US emergency departments which showed that of all emergency visits between 2006 and 2014 due to AARs among older adults (≥65), less than 10% were related to anaphylaxis.^13^ The total number of people suffering from non-anaphylactic versus anaphylactic AARs in Sweden is not established. However, as a rough indication, the prescription of betamethasone soluble tablets, which is the primary corticosteroid prescribed for allergic reactions in Sweden (main indication: short-term treatment of inflammation and allergic reactions), was prescribed to ≈160,000 adults per year in Sweden between 2018 and 2021. In comparison, only 30,000 adults had prescription for epinephrine. These numbers indicate, consistently with our study sample, that there may be a relatively large population of allergy patients in Sweden falling into the category of moderate to severe non-anaphylactic AARs.

An apparent strength of this study is the capturing of the patients’ perspectives. Moreover, the study included a broad representation of people with experience of AARs of different severity representing a range of allergy types and symptoms. Another strength is the stratification based on prescription for epinephrine, which provided an opportunity to separately characterize people with moderate to severe non-anaphylactic reactions. However, it should be noted that an epinephrine prescription does not provide a clear-cut division between respondents with different degrees of severity of AARs, or between those with and without a history of anaphylaxis. International studies have reported an underuse of epinephrine for treatment of anaphylaxis in health care,^14^ including limited prescription of epinephrine.^15^ Moreover, in 2022, the prescription rate for epinephrine varied between 1.9 and 4.7 persons per 1000 inhabitants above 20 years in different Swedish regions.^16^ This likely reflects under-prescription of epinephrine in some regions, and possibly prescriptions of epinephrine as a measure of safety also for patients with experience of less severe AARs in other regions. In our results, the relatively large proportions of the CS-only subgroup that had used emergency care for an AAR (62%) and been treated with epinephrine (22%; presumably in emergency care) indicated that there may be a considerable number of people with severe AARs who could benefit from a prescription for EAI.

One limitation of the study was that the recruitment was performed using a web-panel and social media, and hence was not population-based. An implication of this is that generalization outside the study sample is limited and there might be a self-selection bias. As an indication of selection-bias, the study sample included mostly females (80%) and lacked respondents older than 74 years old. The sample also had a large proportion of highly educated people; the proportion (65%) of respondents with a university education was high compared to the general Swedish population in which 40% in the age group of ≥35 have a 3-year university education.^17^ The clinical implications from this study should therefore be interpreted carefully and valued as an explorative analysis of the disease burden of moderate to severe AARs from a patient perspective.

## Conclusion

Our investigation showed that moderate to severe AARs can have a large impact on daily life in the form of interrupted daily life activities and contribution to anxiety. This was also true for the larger proportion of respondents without prescription for EAI. We also identified that many of the responders had experienced difficulty in swallowing their allergy medicine and not having the medicine available when needed.

## Abbreviations

AAR: acute allergic reaction; CS: corticosteroids; EAI: epinephrine autoinjector; NRS: numeric rating scale; IQR: interquartile range; SD: standard deviation.

## Funding

The study was financed by AccuCort AB.

## Availability of data and materials

Any data or materials supporting the findings of this study, which are not included in the main article or supporting information, are available from the authors upon reasonable request.

## Author contributions

Conceptualization (EA, SL, SO, LB, GT, CJ, JH), study design (EA, SL, SO, JH) data acquisition (EA, SL), data analysis (EA, SL), interpretation (EA, SL, JH, KW), drafting the manuscript (KW, EA), critical review and revision of manuscript (EA, SL, SO, KW, LB, GT, CJ, JH), approval of final manuscript version (EA, SL, SO, KW, LB, GT, CJ, JH).

## Ethical approval and patient consent

The study was approved by the Swedish Ethical Review Authority (Dnr 2022-02147-01). Informed consent was collected from all participants.

## Authors’ consent for publication

All the authors have approved the final version of the manuscript and given their consent to publish in World Allergy Organization Journal.

## Declaration of competing interest

GT is member of the board of AcuCort AB.
