# Supplementary material for: Disease burden and unmet need for acute allergic reactions – A patient perspective
Source: World Allergy Organ J. 2024 Mar 30;17(4):100896. doi: 10.1016/j.waojou.2024.100896 (PMC10990897; doi:10.1016/j.waojou.2024.100896)
Supplement: Multimedia component 1 [file mmc1.docx]

**Supplemental material: Disease burden and unmet need for acute allergic reactions – A patient perspective. Anderson et al.**

# Supplemental Tables

*Supplemental Table S1. List of question from questionnaire survey for which results are presented in the current study. The questions are translated from the original in Swedish.*

| **Questions** | **Answer alternatives** |
| --- | --- |
| **Have you ever had an acute allergic reaction resulting for which you have taken, or been prescribed corticosteroid tablets (**e.g.  **dexamethasone, betamethasone (Betapred), prednisolone?** |  |
|  | Yes |
|  | No |
| Your age |  |
|  |  |
| Sex |  |
|  | Woman |
|  | Man |
| **Which is your highest completed education?** |  |
|  | Compulsory school |
|  | Upper secondary school |
|  | University <3 years |
|  | University ≥3 years |
|  | Other |
| **Which is currently your main occupation?** |  |
|  | Employed (full or part time) |
|  | Have my own company |
|  | Retired |
|  | Student |
|  | Unemployed |
|  | On sick leave |
|  | Other |
|  | Prefer not to answer |
| **What are you allergic to? Multiple alternatives are possible.** |  |
| Food (e.g. nuts, gluten, lactose) | Yes/not chosen |
| Fur (e.g. cat fur, dog fur) | Yes/not chosen |
| Insect stings (e.g. bee stings, wasp stings) | Yes/not chosen |
| Other | Yes/not chosen |
| **Which symptoms do you usually experience in an acute allergic reaction? Multiple alternatives are possible** |  |
| General symptoms (e.g. pronounced fatigue, restlessness, anxiety) | Yes/not chosen |
| Skin symptoms (e.g. itching, rash, itchy eyes) | Yes/not chosen |
| Respiratory (e.g., itching, swelling, cough) | Yes/not chosen |
| Abdominal (e.g., nausea, diarrhea, cramping) | Yes/not chosen |
| Circulatory (e.g., cold sweat, paleness, irregular pulse) | Yes/not chosen |
| Other | Yes/not chosen |
| **How many acute allergic reactions that required treatment with corticosteroid have you had in the recent year?** |  |
|  | None |
|  | 1 |
|  | 2 |
|  | 3 |
|  | 4 |
|  | 5 |
|  | 6 |
|  | 7 |
|  | 8 |
|  | 9 |
|  | 10 or more |
| **How many corticosteroid tablets did you take at your most recent acute allergic reaction?** |  |
|  | Färre än 5 |
|  | 5–10 |
|  | 11–15 |
|  | 16–20 |
|  | Fler än 20 |
| **Have you been treated with epinephrine for an acute allergic reaction?** |  |
|  | Yes |
|  | No |
|  | Do not know |
| **Have you been prescribed an epinephrine autoinjector?** |  |
|  | Yes |
|  | No |
|  | Do not know |
| **How severe was you most recent acute allergic reaction?** **Put a mark on the scale from 1 to 10.** **0 means very mild and 10 means very severe.** |  |
| **Have you ever had emergency care due to an acute allergic reaction?** |  |
|  | Yes |
|  | No |
|  | Do not know |
| **How secure do you feel with your current allergy treatment? Put a mark on the scale from 1 to 10. 0 means very insecure and 10 means very secure.** |  |
| **Are you worried about having an acute allergic reaction? Put a mark on the scale from 1 to 10. 0 means very worried and 10 means not worried at all.** |  |
| **Have you ever experienced an allergic reaction that led to any of the following:** |  |
| Interruption of or inability to carry out daily activities (e.g. work/studies, leisure activities) |  |
|  | Yes |
|  | No |
|  | Do not know |
| Made you fear for your life. |  |
|  | Yes |
|  | No |
|  | Do not know |
| **My allergy limits me in my work/studies** |  |
|  | Disagree completely |
|  | Disagree to some extent |
|  | Agree to some extent |
|  | Agree completely |
|  | Do not know/not applicable |
| **I have had to take sick leave often because of my allergy** |  |
|  | Disagree completely |
|  | Disagree to some extent |
|  | Agree to some extent |
|  | Agree completely |
|  | Do not know/not applicable |
| **My allergy hinders me from participating in leisure activities** |  |
|  | Disagree completely |
|  | Disagree to some extent |
|  | Agree to some extent |
|  | Agree completely |
|  | Do not know/not applicable |
| **Have you ever experienced not having your allergy medicine immediately available when needed for an acute allergic reaction** |  |
|  | Yes |
|  | No |
|  | Do not know |
| Follow up question: For what reason(s) did you not have your allergy medication available when you needed it? |  |
|  | I had not bought/collected my allergy medicine |
|  | I was away from home without my allergy medicine |
|  | Other |
| **Have you ever experienced difficulty in swallowing your allergy medicine when having an acute allergic reaction?** |  |
|  | Yes |
|  | No |
|  | Do not know |

*Table S2. Detailed output data from statistical analyses*

| **Question** | **Test** | **P-value** | **Pearson chi2** | **Z-value** | **T-value** | **Degrees of freedom** |
| --- | --- | --- | --- | --- | --- | --- |
| ***Which is your highest completed education?*** | Pearson’s chi square test | 0.001 | 20.5519 |  |  |  |
| ***What are you allergic to?*** |  |  |  |  |  |  |
| *Food* | Pearson’s chi square test | 0.002 | 9.9711 |  |  |  |
| *Insect stings* | Pearson’s chi square test | 0.003 | 9.3807 |  |  |  |
| *Fur* | Pearson’s chi square test | 0.006 | 8.0348 |  |  |  |
| ***Which symptoms do you usually experience in an acute allergic reaction?*** |  |  |  |  |  |  |
| *Respiratory* | Pearson’s chi square test | 0.002 | 9.1931 |  |  |  |
| *Gastrointestinal* | Pearson’s chi square test | 0.013 | 6.8423 |  |  |  |
| *Circulatory* | Pearson’s chi square test | <0.001 | 33.2903 |  |  |  |
| *General symptoms* | Pearson’s chi square test | 0.120 | 2.6477 |  |  |  |
| ***Median number of organ systems affected*** | Wilcoxon rank-sum | <0.001 |  | -3.437 |  |  |
| ***Have you been treated with epinephrine for an acute allergic reaction?*** | Pearson’s chi square test | <0.001 | 96.1969 |  |  |  |
| ***How severe was you most recent acute allergic reaction?*** | Student’s t-test | <0.001 |  |  | -4.9581 | 385 |
| ***Have you ever had emergency care due to an acute allergic reaction?*** | Pearson’s chi square test | <0.001 | 20.0590 |  |  |  |
| ***Have you ever experienced an allergic reaction that led to interruption of or inability to carry out daily activities?*** | Pearson’s chi square test | 0.002 | 8.9179 |  |  |  |
| ***How secure do you feel with your current allergy treatment?*** | Student’s t-test | 0.827 |  |  | -0.2188 | 385 |
| ***Are you worried about having an acute allergic reaction?*** | Student’s t-test | 0.002 |  |  | 3.1612 | 385 |
| ***Have you ever experienced an allergic reaction that made you fear for your life?*** | Pearson’s chi square test | <0.001 | 56.8153 |  |  |  |

*Table S3. Results from subgroup analysis of respondents with pollen allergy only compared to rest of the study sample.*

| Symptoms of an acute allergic reaction (more than one answer possible) (yes,%) | N=387 | n=39  (only pollen) | p-value |
| --- | --- | --- | --- |
| General (e.g., fatigue, anxiety) | 38 | 51 | 0.124 |
| Skin (e.g., itching, redness, itchy eyes) | 78 | 51* | 0.001 |
| Respiratory (e.g., itching, swelling, cough) | 80 | 62* | 0.014 |
| Abdominal (e.g., nausea, diarrhoea, cramping) | 22 | 3* | 0.003 |
| Circulatory (e.g., cold sweat, paleness, irregular pulse) | 28 | 10* | 0.020 |
| Other | 6 | 0 | - |
| **How severe was your last acute allergic reaction? (Scale from 0-10, where 0=Very mild, 10=Very powerful)** |  |  |  |
| Mean (S.D.) | 7.1 (1.9) | 6.6 (1.6) | 0.083 |
| Median (Q25, Q75) | 7 (5, 7) | 7 (6, 7)* | 0.023 |
| **Experience of an acute allergic reaction that led to interruption of or inability to carry out daily activities (e.g. work/studies, leisure activities) (%)** |  |  |  |
| Yes | 85 | 64* | 0.001 |
| **Experience of an acute allergic reaction that left you fearing for your life (%)** |  |  |  |
| Yes | 43 | 21* | 0.003 |
| **Have you ever had emergency care due to an acute allergic reaction? (%)** |  |  |  |
| Yes | 70 | 54* | 0.035 |
| **Treatment with epinephrine injection for an acute allergic reaction, (%)** |  |  |  |
| Yes | 39 | 13* | 0.001 |
| **Have you been prescribed an epinephrine injection pen? (%)** |  |  |  |
| Yes | 33 | 5* | <0.0001 |
| **How many cortisone tablets did you take the last time you had an acute allergic reaction?** |  |  |  |
| Mean (S.D.) (cortisone tablets) | 8.1 (5.1) | 4.6 (4.6)* | <0.0001 |
| Median (Q25, Q75) (cortisone tablets) | 7.5 (2.5, 13) | 2.5 (2.5, 2.5)* | <0.0001 |

SD: Standard deviation; Q25: 25th quartile; Q75: 75th quartile

*p-value<0.05

# Supplemental Figures

| 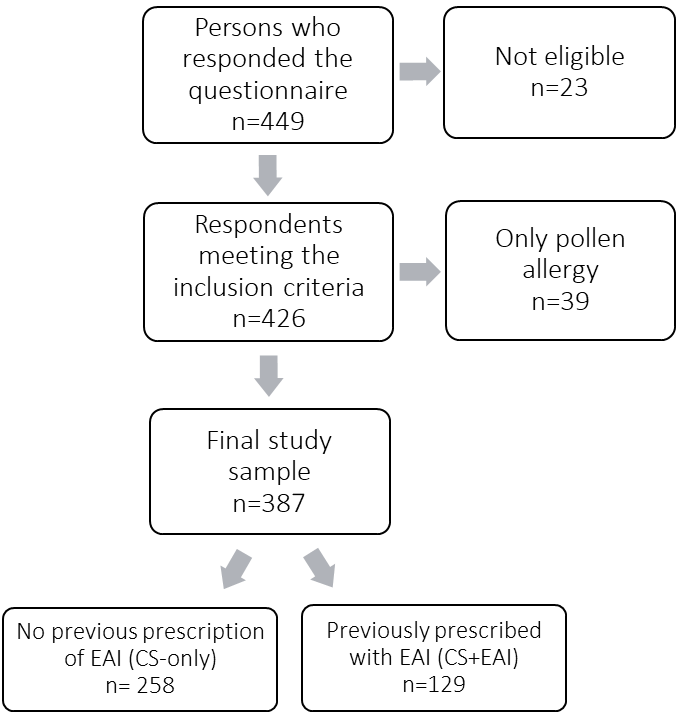 |
| --- |

*Supplemental Figure S1. Flowchart of study inclusion. CS: corticosteroids; EAI: epinephrine autoinjector*

*Supplemental Figure S2. Severity of the most recent allergic reaction, uncombined scores.* *CS: corticosteroids; EAI: epinephrine autoinjector.*

*Supplemental Figure S3. Interruption of daily life. All answer alternatives. CS: corticosteroids; EAI: epinephrine autoinjector.*

*Supplemental Figure S4. Security with treatment and worry about having an acute allergic reaction.* *AAR: acute allergic reaction; CS: corticosteroids; EAI: epinephrine autoinjector.*
